# From Concept to Bedside: What Pediatricians Should Know about Synthesis of Clinical Practice Guidelines?

**Published:** 2014-07-20

**Authors:** Sarar Mohamed

**Affiliations:** Department of Pediatrics, College of Medicine, King Saud University, Riyadh, Saudi Arabia

**Keywords:** Clinical Practice Guidelines, Development, Synthesis, Adaptation, Implementation

## Abstract

Clinical Practice guidelines (CPGs) have emerged as a potentially effective intervention in delivering a high quality, consistent, safe and evidence-based health care. CPGs can either be developed by de novo synthesis or by adaptation of existing guidelines formed in another organization. Guideline recommendations are formulated based on strength of the evidence, validity, clinical relevance and patient values. Support of the organization leadership, role modeling of senior staff and involvement of stakeholders is a key to the success of implementation of guidelines. This article aims to enhance a practicing pediatrician's understanding of how guidelines are developed, disseminated, and potentially utilized.

## Introduction

In the last two decades, there has been an enormous interest among heath care providers, policy makers as well as patients in clinical practice guidelines (CPGs) as a tool for knowledge translation into bedside practice^.[^^1^^-^^3^^]^ .CPGs intend to facilitate consistent and safe heath care delivery that leads to quality improvement^[^^2^^]^. The elements of CPGs development and implementation include assessment of the need of the organization, review of current practice experience in the institute and prioritize topics that require guidelines in place. Drafting of guidelines by an experienced multi-disciplinary panel after a systematic review of literature to appraise and select recommenddations, is the cornerstone of the guidelines development. Dissemination and implementation of the drafted guidelines needs a program surveillance to track variances and provide feedback to participants, periodic review with assessment of value added, and program modification whenever necessary. This review article aims at providing the practicing pediatrician with an insight into these processes. 

***Definition of Clinical Practice Guidelines***

Reflecting on the existing literature and the growing recognition of the importance of guidelines, professional CPG-constructing organizations focus on standardizing terminology. In 2011, The Institute of Medicine of United States defined CPGs as: “statements that include recommendations intended to optimize patient care that are informed by a systematic review of evidence and an assessment of the benefits and harms of different care options”^ [^^4^^]^. While broadly similar to CPGs, Clinical Pathways (CPs) differ by being more conscious about the sequence, timing, and provision of interventions^[^^5^^,^^6^^]^. In fact the vast majority of CPs are extracts from CPGs. On the other hand, clinical protocols are used to outline the management steps for a single clinical condition. Clinical protocols are more precise, specific and have little scope of variation compared with CPGs^[^^2^^,^^6^^]^. 

***Benefits of CPG***

The ultimate goal of CPGs is to enhance the quality of care and improve patient outcome indicators^[^^7^^-^^11^^]^. This is accomplished by adopting evidence-based recommendations and facilitating the delivery of efficient medical care that closes the gap between research and practice^[^^12^^-^^17^^]^. As care becomes more standardized, CPGs often lead to a reduction in health care expenditure, particularly if the CPGs support improved efficiency of care and better organization of hospital contracting^[^^2^^,^^8^^,^^18^^]^. CPGs also provide a mechanism by which healthcare professionals can be made accountable for their clinical activities^[^^6^^]^. It may be argued that CPGs discourage independent clinical judgment and limit the autonomy of physicians. In fact, the evidence-based culture promoted by CPGs encourages physicians to consider the degree to which their current practice choices align with the best available evidence, instead of depending on expert opinion or anecdotal training biases^[^^5^^,^^6^^,^^19^^,^^20^^]^. Ultimately the decisions, which are made by physicians who utilize CPGs, remain autonomous. There is always an option to remove patients from clinical pathways or make independent decisions within the guideline. Such variations in care should, however, be documented as variances and reviewed when the CPG is updated.

***How CPGs are developed?***

CPGs are developed by either de novo guideline synthesis or by adaptation of existing guidelines^[^^13^^]^. It is critical for organizations intending to develop guidelines to choose between these two methods depending on the availability of resources and expertise.

***De novo development of CPGs***

De novo guideline development is time and labor intensive. A guideline development group (GDG) essentially starts from scratch and performs all of the necessary steps to initiate and implement a unique guideline for a specific setting. The first step in CPG development is the identification of a clinical process or condition for which a CPG has the potential to improve care in an important and lasting way. This going on line with the Institute of Medicine (IOM) recommendation, which prioritizes quality improvement initiatives according to their impact, improvability, and inclusiveness^[^^4^^]^. Careful consideration should be given to the problem being addressed and the likelihood that the proper development and implementation of CPGs will translate into tangible results in mitigating or alleviating the problem for a large number of patients. Once that is considered, attention can turn toward assembling the GDG. The GDG should be multidisciplinary and balanced, comprising a variety of methodological experts and clinicians, and populations expected to be affected by the CPG. This group consists of all stakeholders involved in the clinical care or administrative support of patients with a specific condition, such as physicians, nurses, ancillary staff, policy makers, and support groups. The GDG prepares the scene by providing access to resources and expertise and defines the clinical question(s) that will be addressed by the proposed guidelines. Well-built clinical questions are the key to evidence-based decisions. PICO (Problem, Intervention, Comparison, Outcome) or PIPOH (Population, Intervention, Provider, Outcome, Health center) formats are usually used to assemble these questions^[^^13^^,^^21^^]^. Table 1 shows an example of using PIPOH for defining the clinical question related to development of clinical practice guidelines for management of acute asthma in children in order to search for the available evidence. After defining the clinical question using PIPOH or PICO format, the next step is undertaking a comprehensive literature search.

**Table 1:** PIPOH concept for defining clinical question with an example on childhood asthma management

**Population**	**to which guidelines are intended**	**children less than 15 years**
**Intervention**	that is considered for patients	salbutmol and steroid
**Professionals**	by whom guidelines will be used	pediatrician and nurses
**Outcome**	including patient and health indicators	length of emergency room stay and readmission
**Health care setting**	where guidelines will be implemented	health center

Sources that are commonly used to conduct literature searches include the National Library of Medicine’s Medline database, PubMed, and the Cochrane Library^[^^22^^,^^23^^]^. CPGs databases such as Guideline International Network (GIN), National Guideline Clearinghouse (NGC), Scottish Intercollegiate Network (SIGN), National Institute for Health and Care Excellence (NICE) and Trip Database constitute the main sources of existing guidelines^[^^7^^-^^10^^,^^24^^]^. Searching multiple databases increases the likelihood of a comprehensive review using a combination of both MeSH terms and TEXT words will identify a pool of articles on the guideline topic. Selected articles then undergo meticulous critical appraisal to formulate evidence-based recommendations that form the backbone of the proposed CPG^[^^25^^]^. Different score systems and models for assessment of level and grading of evidence are used to assess the quality and validate the recommendations^[^^26^^-^^28^^]^. Varying levels of evidence will be identified in the literature review. There is a pyramid of prioritization of these levels of evidence ranging from expert opinion at the bottom level and increasing progressively through case reports, case series, case control studies, cohort studies, randomized control trials, and eventually leading to the top of the evidence pyramid which is occupied by meta-analysis of a series of well designed randomized controlled trials through systematic review.

 The Grading of Recommendations Assessment, Development, and Evaluation (GRADE) instrument is developed by the GRADE Working Group that assesses both the quality of the evidence and the strength of the recommendation^[^^29^^-^^32^^]^. GRADE has widely been perceived as having a more rigorous development process for the grading of evidence and recommendations, with adequate description of the quality, quantity and consistency of the evidence^[^^33^^]^. Currently, more than 65 national and international organizations have adopted the GRADE approach ^[^^34^^]^. Recently, an expansion of GRADE system was proposed to make it more usable and reproducible^[^^31^^]^. Guidelines recommendations are then formulated based on consistency, clinical relevance, validity and the strength of the evidence. These recommendations are put together in a CPG draft that is reviewed by peer reviewers as well as other stakeholders including patient support groups. This draft should be pilot-tested prior to its dissemination and publication.

***Guideline adaptation***

Guideline adaptation is a systematic approach to the endorsement and/or modification of a guideline produced in one cultural and organizational setting for application in a different context^[^^6^^,^^13^^]^. It is simply a mechanism by which a guideline is produced by an institute and implemented with or without modifications in another institute. This process is referred to as “adoption” when no changes were added to the mother CPGs. To adopt a CPG, it has to be evidence based, clear, usable, and accessible. Adaptation of CPGs may entail customizing existing guidelines to suit the local context. Adaptation reduces duplication of effort and takes advantage of existing guidelines especially for organizations where resources are limited. 

 ADAPTE collaboration is an international collaboration of researchers, guideline developers, and guideline implementers who aim to promote the development and use of clinical practice guidelines through the adaptation of existing guidelines^[^^13^^]^. The ADAPTE collaboration has developed a manual and a framework for adaptation of guidelines. This framework is divided into three phases: set up, adaptation and finalization (Table 2). In the set up phase, the organization prepares the scene for the process of adaptation. This includes establishing an organizing committee, identifying skills and resources needed, and outlining priority topics required clinical practice guidelines^[^^6^^,^^13^^]^. The adaptation phase of ADAPTE instrument deals mainly with the methodology of adaptation namely search for existing guidelines and critical appraisal of these guidelines to select one of them for adaptation^[^^6^^,^^13^^]^. The main sources of CPGs that require intensive search include: Guidelines International Network (G-I-N), National Institute for Health and Care Excellence (NICE), Scottish Intercollegiate Network (SIGN), National Guideline Clearinghouse (NGC), and PubMed.

***Assessment of guidelines***

After selection of existing guidelines through searching of databases, the guidelines undergo extensive review and assessment regarding their quality, currency, content, consistency, acceptability and applicability^[^^6^^,^^13^^]^.

***Appraisal of guidelines***

The Appraisal of Guidelines for Research and Evaluation (AGREE) instrument was developed in 2003 by a group of guideline developers and researchers aiming at forming a tool for assessing the quality and methodology of CPGs^[^^11^^]^. This tool has been developed initially in English and currently translated to many languages. The AGREE format was refined and updated in 2009 forming AGREE 11 instrument, which is widely accepted and endorsed by international health care organizations^[^^12^^,^^15^^-^^17^^]^. The AGREE 11 instrument contains 23 key items categorized in six domains (Table 3) ^[^^12^^,^^15^^,^^16^^,^^17^^]^. Each domain is intended to cover a separate dimension of guideline quality. Guideline developers are required to declare conflict of interest, which may be potential source of bias. Therefore, policies to encourage reporting conflict of interest by guideline developers are needed to decrease associated bias^[^^35^^-^^37^^]^.

***Scoring of guidelines***

When more than one CPG is identified to be relevant for implementation, the need for an objective tool to choose the best guideline arises. AGREE 11 invented a rating scale for each of its 23 items. The scale is from 1 to 7^[^^12^^,^^15^^-^^17^^]^. Rate of 1 indicates that the item is poorly reported while score of 7 shows that the item is exceptionally reported and all criteria are covered. At least two appraisers independently assess and score guidelines. The average score of the guidelines is calculated according to a known formula^[^^12^^,^^15^^-^^17^^]^. Following the overall assessment of the appraisers, the guideline is accepted for implementation with or without modifications or otherwise rejected. Recently, an electronic calculator of AGREE 11 rater has been developed by McMaster University^[^^38^^]^. 

**Table 2:** Summary of ADAPTE process

**Phase**	**Task**
**Set up phase**	Prepare for ADAPTE process
**Adaptation phase**	1. Define health question
	2. Search and screen guidelines
	3. Assess guidelines
	4. Decide and select
	5. Draft guideline report
**Finalization phase**	1. External review
	2. Plan for future review and update
	3. Produce final guideline

**Table 3:** Domains and items of AGREE 11 instrument

**Domain**	**Number of items**	**Quality issues covered**
**1. Scope and purpose**	3	Highlights the aims of the guideline, its specific health question and the population the guideline intended to target.
**2. Stakeholder involvement**	3	Examines to which extend the guideline represent the views of the intended users.
**3. Rigor of development**	8	Covers the methodology of guideline development and how the recommendation is synthesized.
**4. Clarity and presentation**	3	Investigates the format and language of the guidelines.
**5. Applicability**	4	Discusses the implication of implementation of the guideline on the organization.
**6. Editorial independence**	2	Examines the independence of recommendation and of conflict of interest.

 If a decision is taken to accept a CPG with modifications, a thorough literature search and assessment of strength and level of evidence of amended recommendations is undertaken.

 Accepted CPG will then be reviewed by external reviewers including: policy makers, experts in the field and patient groups. A final report of CPG is drafted after pilot testing for target users. Published guidelines required review and updating every few years. Update explores the controversial issues and difficulties seen during the implementation of the guidelines and suggests changes to deal with these points. When reviewing guidelines, a fresh, detailed literature review is undertaken aiming to add new emerging evidence into the updated guidelines. A recent systematic review evaluated the quality, methodlogy, and consistency of recommendations of CPGs illustrated that more effort is needed to improve the quality of guidelines in order to improve health outcomes^[^^39^^]^.

***Guideline Implementation***

Development of CPG is a lengthy and time-consuming process. It requires a lot of resource allocation in addition to commitment of involved personnel in order to yield a simple, concise and easy to follow guideline^[^^40^^-^^44^^]^. Despite this, an even greater amount of effort will be required during the dissemination and implementation of the CPGs. Once a guideline is finalized an implementation committee needs to be formed by the organization. The role of the committee is to increase the awareness of the staff and try to get everybody on board. Regular seminars, lectures and small group discussions with intended users of guidelines cannot be over emphasized^[^^45^^]^. These sessions explain the importance of guidelines; the road map for implementation and the possible challenges may be faced during this long journey. Support of the organization leadership and role modeling of senior staff is a key to the success of implementation of guideline^[^^18^^,^^46^^]^. Good communications with stakeholders, meticulous follow up of implementation details improves adherence to and compliance with guidelines ^[^^5^^,^^47^^,^^48^^]^. Demonstration of the positive changes and closing the loop by auditing and re-auditing improves the outcome of guidelines^[^^49^^]^. Adherence to the guidelines can be improved by anticipating and overcoming organizational and system level barriers^[^^50^^,^^51^^]^. Providing active feedback to the stakeholders on a recurring basis will allow the demonstration of a positive change, which has occurred following implementation. This also allows the team to close the loop by reporting the results of program audits regarding the level of compliance with the guidelines. 

 Utilization of an electronic medical record (EMR) and computerized physician order entry (CPOE) with specific order sets can improve physician compliance in settings where this technology exists. In certain circumstances, decision support tools may be embedded in the electronic medical record as a reminder to physicians when they should consider placing specific orders, which are triggered by specific criteria^[^^52^^]^. Utilization of computerized clinical guidelines (CCG) might improve the compliance of physicians and advance implementation and outcome of guidelines. Implementation of CCG may be integrated with more training and investment in user-friendly hardware and software^[^^52^^,^^53^^]^. This process places the evidence in the hands of providers at the site of care by embedding electronic order sets for treatment and medications using available guidelines. This also enhances implementation strategies by increasing the awareness of staff, facilitating easy access to evidence and encouraging feedback from stakeholders^[^^3^^]^. Similarly, involving patients and the public in development and implementation of guidelines has a tremendous effect on the yield of guidelines^[^^54^^-^^56^^]^.

## Conclusion

CPGs are highly important tools for delivering consistent, reliable, and safe care that decrease variation in practice among heath care professionals and promote standardization of care. It is important that physicians have a clear understanding of the entire process of guideline development and implementation in order to achieve tangible and sustained improvement in clinical practice.
